# “Recovery Came First”: Desistance versus Recovery in the Criminal Careers of Drug-Using Offenders

**DOI:** 10.1100/2012/657671

**Published:** 2012-12-30

**Authors:** Charlotte Colman, Freya Vander Laenen

**Affiliations:** Department of Criminal Law and Criminology, IRCP, Ghent University, 9000 Ghent, Belgium

## Abstract

The aim of our paper is to gain insight in the desistance process of drug-using offenders. We explore the components of change in the desistance process of drug-using offenders by using the cognitive transformation theory of Giordano et al. as a theoretical framework. The desistance process of drug-using offenders entails a two-fold process: desistance of criminal offending and recovery. The results however indicate that desistance is subordinate to recovery because of the fact that drug-using offenders especially see themselves as drug users and not as “criminals.” Their first goal was to start recovery from drug use. They were convinced that recovery from drug use would lead them to a stop in their offending. In the discussion, we explore the implications of this result for further research.

## 1. Introduction

Since the early 1990s, the interest in criminal careers has been increasingly reflected in the criminological research. Although there is a longstanding tradition of criminal career studies (onset and duration), the study of desistance from crime (the end of a criminal career) is a more recent research area. Some scholars define desistance as a *termination point* [1, 2], albeit most scholars prefer to see desistance as a dynamic and gradual process because several turning points can occur during the criminal career [3, 4]. When a person experiences life events such as finding a job or getting married, their social capital can increase through entering into those new social bonds [5]. These life events can then be considered as turning points away from crime.

Different theories explain desistance from crime. A key-theory on desistance is the age-graded informal social control (AGISC) theory of Sampson and Laub [5]; a dynamic model to explain the development of the criminal career. The AGISC-theory states that individual changes occur because of the development of social bonds. Social bonds can be considered as stakes in conformity and they act as a reason to stop offending [5, 6]. Social bonds are a dynamic characteristic since the strength of the social bonds can vary over time and can change depending on the age of the individual, making it an *age-graded informal* social control theory [7–9]. Furthermore, Sampson and Laub acknowledge the importance of human agency as a central element in understanding crime over the life course. They see individuals as active agents, engaged in transformative action oriented towards their future self (e.g., as a “desaster from crime” or as a “family men”). They have the *choice* and *individual will* to give up crime.

Maruna [4, 10–12] elaborates on agency in his “narrative perspective.” According to Maruna [4], desistance occurs when the intrinsic motivation to change (inner change agent) is present [4, 13–15]. To desist from crime, (ex-)offenders need to develop a prosocial identity for themselves. Maruna makes a specific distinction between a *condemnation script* (story of the persisters) and a *redemption script* (story of desasters) [4, 16].

Next to Maruna, Giordano and her colleagues focus on agency and in particular on the role of the actor in the change process. In their cognitive transformation theory, they introduce the concept of *cognitive shifts* as part of the desistance process [17]. Based on the theory of symbolic interaction, they stress that human agency requires *choice and power*. In this context *hooks for change*, that is, turning points, can serve as a catalyst for change. Giordano et al. indicate that the desistance process consists of four steps. The first step is an openness to change; the offenders need to realize that change is necessary and desirable. This requires reflection and reassessment. Second comes the exposure to the *hook for change*, the opportunity to change. The third step is an insight in the conventional “replacement self,” the possibility to see themselves in the new role. The fourth and final step is the transformation away from criminal behaviour and the consideration that the former behaviour is negative [2]. The first and second steps focus on openness and willingness to change and the necessity to answer to the opportunities to change. The third and fourth steps are related to the development of a new conventional identity. Individuals need to have the ability to recognize and to show their openness for that *hook*. This however requires agency: the desire, the ability, and the access to change [18].

The desistance research has regularly developed theoretical insights and empirically studied the role of life events, such as marriage [5, 19] and employment [20, 21] as important elements of social control in desistance [22, 23]. However, life course theories have overall left agency out of the theoretical picture [24] and most longitudinal data sets do not provide the researcher with the opportunity to empirically study the role of human agency [24].

Nevertheless, human agency is an important element in the desistance process. For some offenders, life events like marriage and having a job have a positive influence on desistance, while the same life events do not appear to have the same influence on others. However, there is no clarity about which factors play a role in which circumstances. Agency could have a mediating effect on the objective factors that have an influence on desistance. Hence, the motivation of the offender to change and the attitude of the offender towards those social bonds are also crucial.

The aim of our paper is to get insight in the desistance process of drug using offenders by using the cognitive transformation theory of Giordano et al. as a theoretical framework. Firstly the cognitive transformation theory is a widely known and empirically tested theory [25, 26]. Secondly, in this study, we want to further explore the readiness for change and investigate how the desistance process of drug-using offenders works. After all, Giordano et al. refer themselves to the group of drug-using offenders in explaining the first type of cognitive transformation, namely, exposure “*The most fundamental (step), is a shift in the actor's basic openness to change. The importance of this readiness for change has been discussed extensively in various treatment literatures, especially those dealing with addictions*” [17, page 1000].

To this end, this study has one central question: *what are the components of change in the desistance process of drug-using offenders?*

## 2. Method

The current study is part of an ongoing Ph.D. study on turning points in the criminal careers of drug-using offenders. Unraveling the contributing elements in the recovery and desistance processes and the way in which they have influenced each other calls for a qualitative research. Only in such a design can the subjective experiences of drug users be put at the centre of the study in order to increase insight into the “how” and “why” of desistance [4, 27]. The research design comprised of semistructured interviews in which implicit meanings and reflections can be taken up with the respondents [28]. The questionnaire was based on the questionnaire used in the study of Byrne and Trew [14], complemented with the questionnaires of the studies of Rönkä et al. [29] and Laub and Sampson [3].

The current study is aimed at *desisting* drug-using offenders. In order to find individuals who had been strongly involved both in offending and in drug use, the study makes use of gatekeepers in order to identify suitable research subjects. After all, this population can be considered as a hidden population. Gatekeepers were sought in treatment services and in social work services (so-called “street corner services”) rather than in prison staff, because the former are more suited for identifying *desisting* drug users. The gatekeepers were contacted in 13 different cities in Flanders in order to secure adequate territorial coverage. Gatekeepers identified and established the contact with 35 respondents, after which snowball sampling was used to come into contact with 5 additional respondents [30]. The snowball sampling was limited because most respondents broke contact with a former drug-using context.

After the snowball sampling, we conducted a critical case sampling to decide on the inclusion of the respondents for the interviews. The criteria for whether or not to be included depended on the assessment of the gatekeepers and the self-reports with regard to drug use, offending, and desistance. With regard to the use of *illicit drugs*, previous use “on a regular base” was required. To determine which use constituted “regular use,” the definition of Nelles et al. [31] was used, stipulating that drug use is regular when it happens “*at least three times a week for 1 year*” [31]. With regard to *offending*, our criteria were that the respondents had to have committed at least five offences (property, violent, sexual, or consensual crimes) during a period of five years. A minimum of five offences is required in order to select those exoffenders who previously had “criminal careers” and to exclude first and/or occasional offenders. In order to be able to study their *desistance process*, this process needed to have started one year before the inclusion in the research project.

In total, 40 desasters were interviewed, 32 of them male and 8 female. They had desisted from offending for a period of, on average, 28 months. The respondents had used several types of drugs and had committed several offences.

The interviews lasted between one and three hours. Their anonymity and confidentiality were guaranteed. Respondents were informed about the project, first briefly by the gatekeeper, later on in detail by the researcher. All respondents signed an informed consent (describing the research theme, their (confidentiality) rights and a contact address for further information). The interviews were recorded, after the consent of the respondent had been obtained. Afterwards transcripts were made and processed using specific software for qualitative analysis (NVivo) using a codebook. We used the four phases of the cognitive transformation theory as the basis for this codebook and the further analysis.

## 3. Results

### 3.1. Recovery rather than Desistance

The respondents were asked how their desistance processes had evolved, both with regard to drug use and to offending. Starting from the four phases desistance process, described by Giordano, we notice that the desistance and recovery process of drug-using offenders is complex.

For some of the respondents, their offending was limited to selling drugs, without this being dealing at a large scale. Rather they sold drugs to their friends, with no other goal than to obtain some extra money for their personal drug use. Mostly, they never earned a lot of money with it, so for them desistance from offending was easy and a logical result of their drug use desistance, although there could be a lag between the desistance from drug use and the desistance from dealing.

*I was dealing drugs. But it was not dealing, dealing. It was for my own use and as a favour for my friends. Our “gang” that was fun. I did not earn a lot of money with it, so for me it was easy to stop doing it. (Male, 38, desisted for 1 year)*

A significant group of the respondents committed offences in order to have enough money to sustain their own drug use. Their offences were property offences and could be denominated as the so-called acquisitive offences. In these cases, offending only started after the drug use and could be considered a consequence thereof. Because of this, we see that the respondents consider their desistance from offending to be subordinate to their drug use desistance. Their first goal was to stop using drugs. Because both offending and the use of drugs were related to each other, they were convinced that desistance from drug use would lead to a stop in their offending.

*I stole to have money for food and also a bit for drugs. I stopped offending because it was not necessary anymore because I did not have to buy drugs anymore. (Female, 37, desisted for 9 months)*

*For me, stop using drugs and committing offences were related. But to stop using was the most important thing. Because I knew: “if I stop using, then I do not have to offend anymore.” (Male, 39, desisted for 1 year)*

A minority of the respondents was involved in offending in a way which was not strictly related to their drug use. They had had several contacts with the police and with judicial authorities. Unlike the previously mentioned persons, these respondents experienced desistance from offending as a conscious process. They grew to see their involvement in offending as being at odds with their new responsibilities and life styles. They wanted to avoid going to prison, not so much because they fear prison in itself but rather because a stay in prison would jeopardize their lives as a partner or as a parent.

*You have a certain responsibility now. Why don't you want to commit offences anymore? Because you don't want to leave your partner behind on her own. I am not afraid of prison, but I would be afraid of leaving her on her own. It frightens me more than prison. I chose consciously not to commit offences ever again. I had already stopped offending when I stopped using drugs. I don't think that they had a very strong influence on one another. It was the sense of responsibility that made the “click”. (Male, 38, desisted for 4 years)*

To conclude, most of our respondents (four out of five) consider their desistance from offending to be subordinate to their drug use “desistance” (*so recovery*). Their first goal was starting to recover from drug use. They were convinced that recovery from drug use would lead them to a stop in their offending. After all, as seen in the literature, commitment to recovery is related to one's quality of life which in turn can be enhanced by (re)gaining and maintaining certain desired needs in life (e.g., stable housing, education and work, family, well-being, stable financial situation) [32, 33]. In the interviews, they could not answer the question how their desistance process from offending developed. For them, desistance from offending is not a conscious process of making the choice for change, but rather a consequence of their new life style, namely, a drug-free life. As a consequence, for the analysis of the key concepts of Giordano's cognitive theory, *we focus on the recovery from drug use rather than on desistance from crime* in the remainder of the section.

### 3.2. Openness to Change

Several respondents indicated that at a particular time in their lives they “reformed,” they made a change which they describe as a “click.” They found the motivation to change their life. For most respondents the exact cause of that motivation is difficult to identify. They cannot explain what the trigger was to make the decision to stop using drugs. They can only say that they wanted to change themselves and their lives.

*It couldn't last anymore, it was not livable. Waking up, against my will, never fully awake, working, money. Never enough money because you have spend too much on partying. You feel dirty… ultimately, it had been enough. (Male, 25, desisted for 2 years)*

Some of these respondents did refer to a specific cause for their openness to change, when they explicitly mention the “aging”-argument described in desistance literature [34].

*A lot of things happen during your drug-using career. At a certain moment it cannot go on anymore and then you have two choices: continue what you are doing until you die or say to yourself “I am already 55, I do not want to die when I am 56.” (Male, 55, desisted for more than 20 years)*

Most respondents came to the decision to change after evaluating their life course and realizing the need to change. They indicated that they wanted to have a future, that they did not want to remain an outsider and wanted to become an active member in society. For some respondents, this assessment process was concluded after several months, for others this assessment process took years. For most respondents in this group, this reflection started after a difficult period in their life of heavy drug use and drug-related crime. For others, the reflection process started when they experienced the weakening of social bonds, that is, periods when social bonds were at stake or already lost.

*I started shoplifting, I lost my job, I lost my girlfriend, I lost my parents. I had nobody. So I've said to myself: I have to stop. Otherwise, I would have killed myself. (Male, 34, desisted for 2 years)*

### 3.3. Exposure

A key issue in recovery, according to the respondents, is that recovery should be motivated by internal rather than by external reasons, such as the presence of external social bonds. Most respondents place the entire responsibility for recovery on themselves. To them, it is clear that the real turning point with regard to their drug use should be situated in their own decision to stop using, arising from their own motivation. According to the respondents, drug use is intrinsically personal and motivated by the self. Because drug use is so attached to personal—selfish—motivations, recovery should be as well.

However, this does not imply that external factors do not play some role in this process. External factors such as family, relationships, or death of peers can trigger the internal motivation or can provide the added value to make the decision to stop using drugs. Another person or a change in social bonds made respondents realize that change was necessary. Most of the respondents mentioned family and new or changing relationships. Especially starting a new romantic relationship or becoming a parent have been denominated as the most important external factors which led to an internal motivation. In some cases, this immediately led to desistance. In other cases, several years passed before these personal ties led to change, for example, when the relationship was in danger or when they would lose custody of their child. At these moments, they realized what they could lose. Besides family and personal relationships, a large group of respondents mentioned the influence of treatment. Some respondents mentioned difficult periods such as the death of a relative or friend. This period often made the respondents think about their lives. Such strong impacts and emotions made them also push through when they were at risk of a relapse. Finally, a few respondents mentioned the influence of the criminal justice system.

*In treatment I got structure. I knew that I needed that, therefore I also did my best. Without treatment, I could not have done it. It took me a long time to tell myself that it could not go on like that… I went to treatment for my girlfriend… In treatment I did everything to get her back, but it's not because of her that I stopped. I stopped not only for her, but also for me. She is the cherry on top…. Of course she stimulated me, but it was not enough…. I can't stop for somebody else. … (Male, 25, desisted for 2 years)*

Openness to change and exposure to change seem to go hand in hand. Most respondents indicated that it was their decision to stop using and that external factors are of secondary importance. With regard to “*hooks for change*,” it is important that the individual recognizes the *hook* and considers it as a chance to desist from their deviant behavior. This process demands a level of active involvement and the energy to grab the chance to change. Where openness can stand alone and lead to recovery, exposure requires the openness to change. When the respondent does not realize that change is necessary, they could not stay abstinent. Some respondents compared their current recovery process with previous ones, in which they had stopped using for somebody else (e.g., partner, parents…). In their previous attempts, this external motivation for change was not sufficiently bent towards an internal motivation, which led to relapses. It was only after these periods of relapse that they had found a personal motivator for change, which ultimately led to their recovery. This means that external social bonds need to be accompanied by openness to change (the internal motivation).

*My mother used to say that I should stop using for her. But that never worked. I think that when you are doing it for somebody else, that it does not work. You should want it for yourself. When you are doing it for somebody else… at these moments when you are using drugs or ready to use drugs, you do not think about somebody else, you think about yourself. That is why I am doing this for me. You can try to stop for somebody else, but sooner or later you start again, that is what the past has shown me. Now that I am doing this for me, it finally lasts. (Male, 19, desisted for 6 months)*

### 3.4. Insight in the Conventional “Replacement Self.”

Most of the respondents indicated that they wanted to change because they wanted to be themselves again. Like mentioned before, they felt like they became someone else during their drug using period, especially when their drug use was combined with offending—the latter being contrary to their personality (which was also found by Maruna [4] in the redemption script). When recovered, they wanted to have a new role as a father, as a partner, or a nondrug user. The respondents made plans for the future, they realise they wanted to make something out of their lives.

*At forty, I want a house, a job, be somebody in life. I am willing to give it all up to achieve my goal. I do not go out anymore, I have stopped smoking and I am even a sportsman now. First the baseline, the foundations and then the rest… You just have to realize you have to take it step by step… Just continue and fight. (Male, 38, desisted for 2 years)*

Often they compare their situation with that of others, mostly nondrug using people. They regretted they not yet achieved the same things as people in their age group have. But at the same time, it motivates them to make something out of their lives. They assessed what they had achieved and still wanted to achieve on different life domains. The most important life domain for them was family; they wanted to be a good mother/father to their children. Secondly, the development of a stable relationship with their partner was frequently mentioned. Finally, finding a (different) job was also considered to be an important goal. They wanted to develop meaningful relationships (with their children, with a partner), they want to be a good parent, and they wanted a satisfying job. Often, they had lost everything (custody, their job) and the moment they decided to recover, they wanted to regain these lost social bonds and start fighting for it.

*I could no longer carry on like that, it had to end… I had no life… I started comparing myself to my sister: university, married… and what had I achieved in my life? I was jealous of people who had done something with their lives, who took care of their children, worked and owned a house. And now I want the same. (Female, 28, desisted for 3 years)*

### 3.5. Transformation

Looking back on their past and present behaviour, respondents realize that they were “someone else.” This is particularly the case for respondents who combined drug use with acquisitive crimes. Almost all respondents look at their lives differently and have another goal expectation compared to the period in which they still use drugs or committe crimes. Their image of themselves and their personality also changed. When they used drugs, and committed crimes, they were someone else; they feel that their behaviour was contradicting their personality. Looking back at their past behaviour they label themselves as a “junk,” although at that time they did not realize that they were having problems with drug use. Interestingly, none of the respondents labeled themselves as former offenders.

*I never realized it was so bad. I had a job. If you asked me: what is a junk? I thought: it is somebody who lies in the gutter, without work and with a needle in his arm. I never was like that, but still I was a junk. The image I had of a junk was something completely different than me, but still I was one. (Male, 25, desisted for 2 years)*

As mentioned in the former phase (replacement self), the respondents see themselves in their new role before they are actually transformed. Since they are very realistic about their future, taking into account that a former drug user should never consider him/herself to be completely “recovered;” therefore, they do not call themselves recovered persons successfully reached the end of the recovery process, but rather recovering persons, indicating they consider recovery as an ongoing process.

*Yes, it is always difficult. Both physically and mentally. Every day I am tempted. I won't say “I'm clean and this is for the rest of my life”. It's like alcoholics. I am clean today, but we'll see about tomorrow…. For now I am ok. You have to live from day to day, especially with heroin. (Male, 38, desisted for 1 year)*

Although former drug-using offenders are oriented towards the future, they are still to some extent contemplating their past because it is a very important aspect of their recovery process. They consider their past as a kind of life experience, a period that made them think about the direction of their lives and made them want to focus on other goals.

*I have lost a lot of money and I hurt a lot of people who loved me without even realizing because I was so tangled up in the drug scene… I know that you should look ahead and not backwards, but it will always be a part of my luggage. (Male, 34, desisted for 2 years)*

The respondents also indicate that they continue to fight a labeling process. During their drug using period, they were somebody else: a junk, a criminal. Now that they are recovering, they become themselves again, “the clean person.” A difficult obstacle however is that society needs to accept them as a clean person (again) and needs to accept the new roles they are willing to take. This is not always evident. Therefore, some respondents want to move to another city where they can make a fresh start.

*I've changed for myself. I want to be part of a group of clean people. I don't want to be the outsider… the user…the junk… Although they still look and point at me “Look there, a junk!”. That label will last forever… until my death. (Male, 37, desisted for 4 years)*

### 3.6. Behavioural Change

Entering new social environments and (re)establishing social bonds, as well as avoiding or breaking contact with previous networks, is denominated an important element in desistance. To sustain recovery from drug use, most respondents identified that they had to break with a drug-using partner or drug-using friends. They prefer to start a quest for (drug-free) bonds who could support them in a life free of drug use, and consequently, of crime.

*I left it behind me. I broke up contact with everyone (former friends). Otherwise, they would say: Come on X., it would not do you any harm. That's the reason I don't want to see them again. (Female, 41, desisted for 13 years)***

## 4. Discussion

### 4.1. The Cognitive Transformation Theory Is Applicable to Drug Using Offenders

The population of drug-using offenders is of special interest for the study of desistance because of the influence of drug use on the development and continuation of criminal behaviour [35], since this population commits a substantial number of offenses [36] and since recidivism rates are high amongst this population. Thus, drug-using offenders have a bigger chance to develop a long lasting criminal career [37]. From our interviews with desisting drug-using offenders, it became clear that *the cognitive transformation theory and its different phases are applicable to our research group*. Like Giordano and colleagues mention in their cognitive transformation theory [17], it is especially the first phase, openness, and readiness to change that is characteristic for drug-using offenders. Intrinsic motivation is a key factor in the recovery process [38, 39]. Besides that openness, drug-using offenders need the opportunity to change. Their *hooks for change* are mostly relationships, family, and treatment related. It became clear though that most of our respondents are stuck in the third phase, before the identity change (fourth phase). They consider their past behaviour as negative, they want to become themselves again, and they want to show their new role to society. But most of the respondents do not believe that a real transformation is possible since drug addiction is a long lasting problem and since society is still labeling them as such. In fact, they are quite realistic about their success rate, distinguishing them from other groups of offenders. Where other types of desasters make ambitious plans for the future (as became clear from the redemption script described by [4]), drug-using offenders take into account the possibility of personal relapse and societal rejection. They are always alert for situations or people who can tempt them to start using again. This type of ambivalence is widely recognized in desistance literature and is thought to be common in the first stages of change [40].

### 4.2. Desistance Is Subordinate to Recovery

From research, it has become clear that the link between drug use and crime is not straightforward [41]. Researchers have been interested in establishing what came first: drug use or crime. Because of the intertwined relationship between drug use and crime, it is not obvious to distinguish both processes. There are not a lot of studies on the specific desistance process of drug-using offenders. This small amount of studies uses in most cases desistance (from crime) and recovery (from drugs) as synonyms [42, 43]. This study aimed to gain insight in the desistance process of drug-using offenders. We started from the desistance perspective and explored whether a general desistance theory is also applicable to drug-using offenders (it was however not our aim to test this theory). However, during the study respondents indicated that “to them” recovery is more important than desistance. In most cases the respondents indicate that desistance is a result of recovery. However, this does not imply that their goal is abstinence. 15 respondents were still using drugs in the year preceding the interview. Despite their drug use, these respondents identified themselves as desasters; for instance, they were former regular heroin users but since that period they switched to regular cannabis use. We did include these respondents in our study when they identified themselves as desisting persons. In the past, lifelong abstinence was seen as the only indicator of recovery. In recent years, however, abstinence is seen as just one indicator of recovery and not the only or ultimate goal; significant reductions in drug use are also seen as important indicators [44].

### 4.3. The Transtheoretical Theory as an Extension of the Cognitive Transformation Theory

Recovery and desistance are two research traditions originated from a different context, developed parallel to each other, and seldom interconnected. Recovery originated from the mental health discipline; desistance originated from the criminal career tradition and it is predominantly criminological focused. However, theories on recovery and desistance have important similar characteristics. Recovery and desistance are both transformational processes and not linear but dynamic and gradual processes. People in the process of recovery and desistance are active agents. These processes require human agency which in its turn demands individual choice and power. The major difference between recovery and desistance is that has to do with the final “goal” of change. Regarding recovery, the people themselves define what recovery entails. White has described this goal for people living with psychiatric and/or addiction disorders as “*to eliminate or manage their symptoms, increase their capacity to participate in valued relationships and roles, and embrace purpose and meaning in their lives, in other words, experience recovery*” [45]. In desistance, however, the focus is mainly on socially desirable outcomes (e.g., no illegal drug use, no criminal offences, employment) and less on client-reported outcomes and starting from clients' own expectations and experiences (e.g., quality of life) [46]. As active agents, the respondents in our study challenge some of the socially desirable outcome indicators of change, in particular of no drug use. Following the drug users' perspective, when tackling drug-related crime, it is as important to tackle the drug using problem and related problems on other life domains, besides the criminal problem. After all, in most cases, when controlling the drug problem, the criminal career will be positively influenced.

We are thus convinced that these two traditions can learn from each other and that evidence of one tradition can extend knowledge of the other. An illustration of this is the transtheoretical theory of Prochaska [47].

### 4.4. Structural Constraints

We already mentioned the evolution in criminological theories. Hirschi rephrased the question of “why do offenders commit crime” into “why do they not commit crimes,” which led him to explore social bonds and focus less on motivation. The present scholars however acknowledge and emphasise the role of the individual actor. This theoretical shift can be situated within a cultural shift. In the modernization process, our society became more individualistic. At microlevel, individuals act more independently. Some social norms do not exist anymore or became more liquid [48]. More responsibility is given to the individual and less attention is given to his/her social bonds. In this study, we found the same state of mind. Most of the respondents place the entire responsibility for recovery on themselves. For them, it is clear that the real turning point with regard to their drug use should be situated in their own decision to stop using, with self-motivation as a starting point. According to the respondents, drug use is intrinsically personal and motivated by the self. Because drug use is so attached to personal and selfish motivations, recovery should be as well.

In this context, some authors represent the symbolic interaction theory as a perspective that works well for describing individual behaviours, but not the society/group behaviours [49, 50]. Giordano et al. recognise this criticism in their cognitive transformation theory [17]. Intrinsic motivation is important but not sufficient to abstain. The (immediate) social world plays an important role in this regard. It is not because you WANT to change that you will SUCCEED. Structural constraints/barriers for instance the stigma of former drug users or the lack of job opportunities for former prisoners play a significant role [51]. Our respondents noticed these broader social forces by describing the difficulty of living with the label “ex-drug-using-offender.”

Based on our results with desisting drug using offenders, we formulate two recommendations for future desistance research.

Firstly, in most desistance studies, drug use is not studied as a separate element in the desistance process. Drug use is mostly considered as a risk factor in the desistance process of crime [52]. Less desistance studies are focused on the factor of drugs as an inherent factor of the desistance process and the (inter-)relationship between recovery and desistance. This paper however illustrates the importance to see the drug using part as a crucial part in the desistance process. Like mentioned several times, our respondents see themselves as drug users, not as criminals. Future studies on desistance need to emphasise the factor of drug use and consider it as a separate factor instead of a part of antisocial behaviour. This is particularly relevant since research established that 70% of repeated offenders are regular users [36] and 14% of registered crime (property, sexual, and violent crimes) is drug related [53].

Second, when looking at desistance, it is important to look, next to the official reports registering offending, to the perception of the offender/desister itself. If this study was based on official data, more than a half of the respondents could not have been considered as “desasters.” More than 60% of the respondents were still using cannabis at the time of the interviews but they had recovered from heroin use. They do not consider cannabis use as “drug use” as they do not consider dealing, in the form of social supply as a “criminal act” [54]. This also refers to the earlier mentioned state of ambivalence (referring to the fourth phase of the TTM). Although they considered themselves as nondrug users or noncriminal, they could still have had arrest records or convictions relating to their cannabis use or dealing. So, when studying desistance and its underlying processes, it is recommended to include a qualitative study on the perceptions of (former) offenders, next to the study of official police data since the former will add to the insight into the complexity of the desistance process.

To conclude we stress one important study limitation linked to our sampling method. The sample size of this study was limited. Gatekeepers identified and established contact with 35 respondents, after which snowball sampling was used to come into contact with 5 additional respondents [30]. The snowball sampling was limited because most respondents broke contact with a former drug-using context. Furthermore, our sample consisted especially of men instead of women (ratio 32 : 8), but this is a reflection of reality: more men than women are involved in crime and drug use [55, 56] as well as in drug treatment [57, 58]. Therefore, the results of this qualitative study should be interpreted with caution, as the findings might not be transferrable to the total group of drug-using offenders.
